# Abundance and diversity of fungal endophytes isolated from monk fruit (*Siraitia grosvenorii*) grown in a Canadian research greenhouse

**DOI:** 10.1002/pei3.10142

**Published:** 2024-04-02

**Authors:** Li Ma, Janice F. Elmhirst, Rojin Darvish, Lisa A. Wegener, Deborah Henderson

**Affiliations:** ^1^ Institute for Sustainable Horticulture Kwantlen Polytechnic University Surrey British Columbia Canada; ^2^ Elmhirst Diagnostics and Research Abbotsford British Columbia Canada

**Keywords:** abundance, diversity, fungal communities, fungal endophytes, monk fruit, *Siraitia grosvenorii*

## Abstract

Monk fruit (*Siraitia grosvenorii*) is an herbaceous perennial vine of the Cucurbitaceae family cultivated commercially mainly in southern China. There is very little information available about the fungal endophytes in monk fruit. In this study, monk fruit plants were grown from seeds in a research greenhouse at Kwantlen Polytechnic University in British Columbia, Canada to explore the abundance and diversity of their fungal endophytes. Fungal endophytes were isolated from seeds, seedlings, mature monk fruit plants, and fruits, and cultured on potato dextrose agar and water agar media. Isolates were identified by microscopic examination and BLAST comparison of ITS sequences to published sequences in GenBank. At least 150 species of fungal endophytes representing 60 genera and 20 orders were recovered from monk fruit tissues. Non‐metric multidimensional scaling (NMDS) was carried out to explore the similarity of fungal communities among roots, stems, leaves, flowers, fruits, and seeds based on fungal orders. Our study showed that monk fruit plants are a rich source of fungal endophytes with the greatest abundance and diversity in leaves. This work has deepened our understanding of the intricate interactions between plants and fungi that sustain ecosystems and underpin plant health and resilience.

## Graphical abstract

Monk fruit plants are a rich source of fungal endophytes with the greatest abundance and diversity in leaves. This work has deepened our understanding of the intricate interactions between plants and fungi that sustain ecosystems and underpin plant health and resilience. This knowledge can inform strategies for developing climate‐resilient crops and restoring ecosystems in the face of climate challenges and developing more sustainable and eco‐friendly strategies for plant health management.

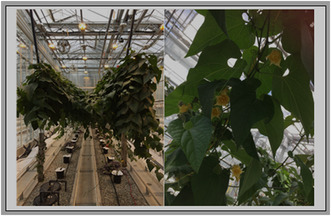

## 1 INTRODUCTION

Monk fruit [*Siraitia grosvenorii* (Swingle) C. Jeffrey ex A.M. Lu & Zhi Y. Zhang] is an herbaceous perennial vine of the Cucurbitaceae family cultivated commercially mainly in the southern parts of China though it is grown also in northern Thailand and has been exported to the USA and India (Shivani et al., 2021). It is commonly grown in Yongfu, Longsheng, and Lingui counties in northern Guangxi Province with an annual average temperature of 16–20°C, average precipitation of 1500–2002 mm, and average sunshine of 1237.3 ~ 1626.4 h (Zeng et al., 2011).

The fruit of the monk fruit vine has been used as natural, calorie‐free sweeteners (Xia et al., 2008) as well as folk medicine in China for thousands of years due to their pharmaceutical properties such as anti‐inflammation (Di et al., 2011), anti‐carcinogenesis (Takasaki et al., 2003), anti‐oxidation, and anti‐obesity (Sun et al., 2012). Mogrosides are the main compounds in the fruit responsible for the medicinal activities and sweetness.

Endophytic fungi live symbiotically within the internal tissues of healthy, living plants. Many are also saprophytic and some species may become pathogenic causing external infections upon plant senescence (Saikkonen et al., 1998; Stone et al., 2000). Most plants in natural ecosystems are hosts to one or more fungal endophytes, which may reside within roots, stems, leaves, and/or other plant parts (Petrini, 1986; Stone et al., 2004). The symbiotic relationship between fungal endophytes and their hosts ranges from parasitism where the endophytes benefit for growth and reproduction at the expense of the host, to mutualism where endophytes confer positive fitness benefits to their hosts while obtaining nutrients for their growth and reproduction (Aly et al., 2011; Rodriguez et al., 2009; Rodriguez & Redman, 2008). Many fungal endophytes have been shown to reduce infection by pathogens or disease development in their hosts (Busby et al., 2016). The transmission of endophytic fungi is primarily horizontal via airborne spores; some however can transmit vertically to new host generations via seed infections (Aly et al., 2011; Saikkonen et al., 2002). Besides their significant impacts on the survival and fitness of plants by conferring stress tolerance, increasing water use efficiency and plant biomass, or decreasing fitness by altering resource allocation (Rodriguez et al., 2009), endophytic fungi also have great potential as a unique source of biologically active compounds with promising applications in medicine, pharmacy, and agriculture (Aly et al., 2010; Nisa et al., 2015; Zhang et al., 2006).

It has been shown that both fungal and bacterial endophytes can modify their genes by absorbing part of the host DNA into their genome for adaptation to the specific microenvironment (Aly et al., 2011; Germaine et al., 2004), which may help explain the ability of some endophytes to produce the same phytochemicals as those produced by their host plants (Stierle et al., 1993). Chen et al. (2020) isolated 15 endophytic fungal strains from roots, stems, leaves, and fruits of *S. grosveno*r*ii* and found that two of them, *Diaporthe angelicae* Berk. Wehm. [syn. *Mazzantia angelicae* (Berk.) Lar. N. Vassiljeva] and *Fusarium solani* (Mart.) Sacc., could produce some of the phytochemicals produced by the host plant. The other endophytic strains isolated from monk fruit were not named in the published report (Chen et al., 2020). There is very little information available about the fungal endophytes in monk fruit. The present study aimed to explore the abundance and diversity of fungal endophytes in monk fruit grown in a Canadian research greenhouse environment, where we can manipulate the environment to mimic the natural cultivating conditions of monk fruit and minimize their interactions with the outdoor environment and potential contaminants. This also avoided the introduction of novel fungal species into the environment.

## 2 MATERIALS AND METHODS

### 2.1 Isolating endophytic fungi from seeds

**FIGURE 1:**
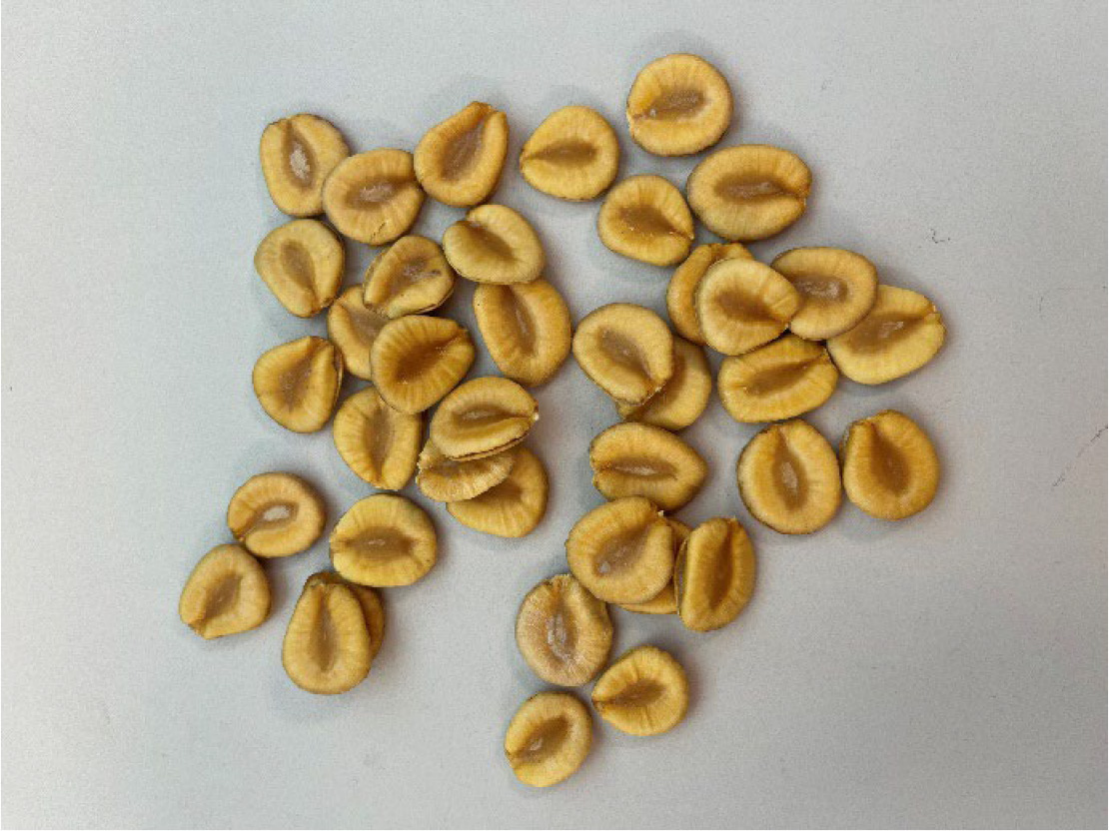
Fresh monk fruit seeds collected from fruits.

In 2020, dry monk fruit seeds obtained via Alibaba from Guangxi Naturix Import & Export Co., Ltd. (Nanning, Guangxi, China) and seeds extracted from commercial fresh fruits (Figure 1) purchased from China. Fungal endophytes were isolated from seeds following the method used by Shearin et al. (2018) with modifications. Seeds were surface sterilized with 10% bleach for 2 min, rinsed with sterile reverse osmosis water three times, and then placed on two types of microbial growth media in petri dishes: potato dextrose agar (PDA) incorporated with 0.005% streptomycin, and water agar (WA) media. The rinse water was plated as a control to ensure that the surface sterilization process was thorough. If fungal colonies were observed in the control plates, the plates were discarded and new seed samples were surface‐sterilized and plated again. Plates were kept in an incubator at 27°C and monitored regularly. All fungal endophytes were recovered from the media and each endophyte was sub‐cultured up to three times until a pure culture was obtained for identification.

### 2.2 Growing plants

**FIGURE 2:**
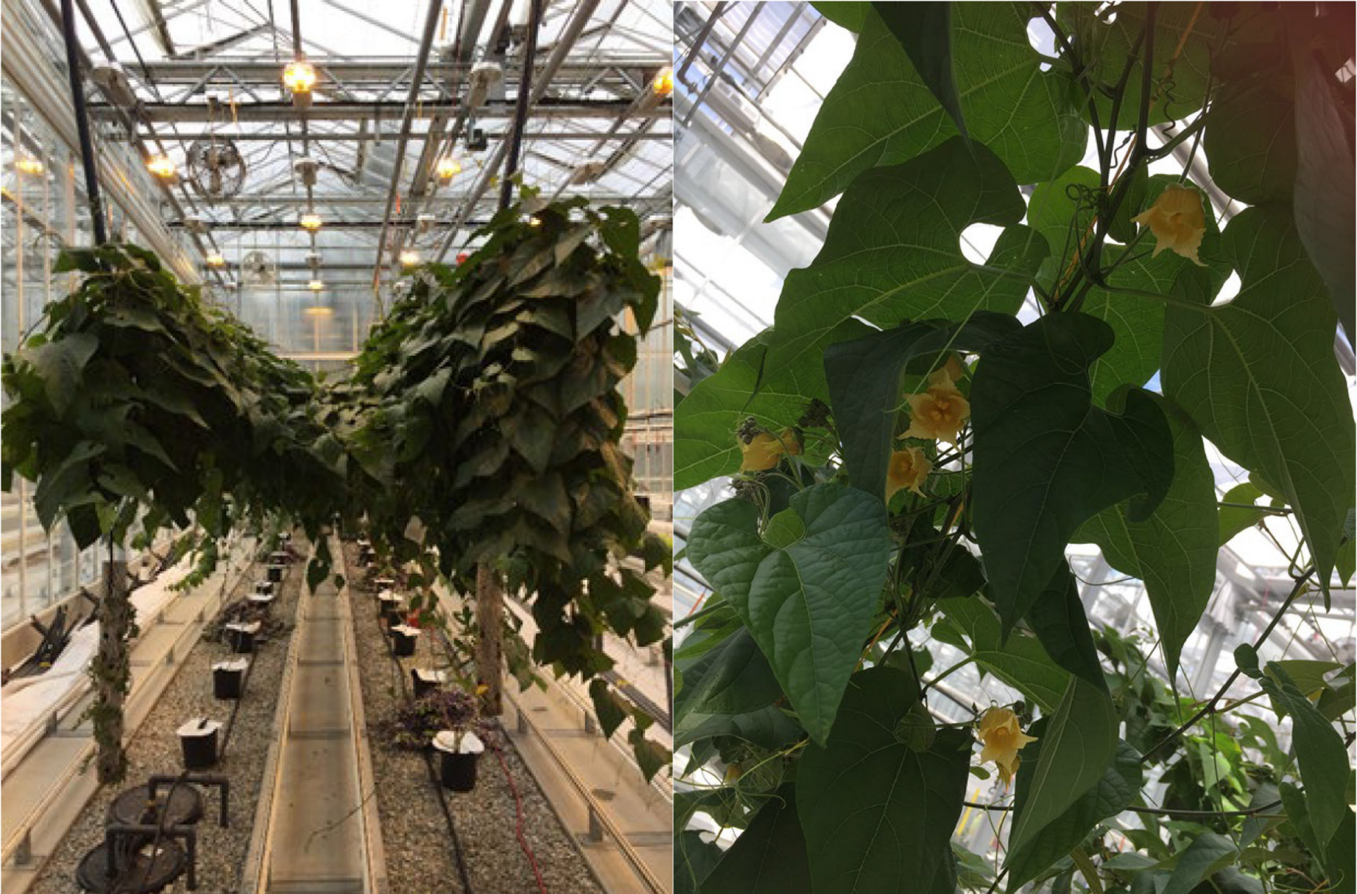
Monk fruit plants grown in the research greenhouse at Kwantlen Polytechnic University, Langley, British Columbia, Canada.

Plants were grown from seeds extracted from the fresh fruit from China. After removing the seed coat, seeds were surface sterilized with 10% bleach and placed on Murashige and Skoog medium in petri dishes to germinate. Seedlings were transplanted into Sunshine Mix #2 potting media in 10 cm (4‐inch) pots and kept in a growth chamber at 21°C and a 16 h light period for 10–12 weeks. After five seedlings were taken for endophyte isolation at 9–10 weeks, the remaining seedlings were transplanted into Sunshine Mix #4 in 15 cm (one‐gallon) pots, one plant per pot, and placed in the research greenhouse located on the KPU Langley campus in January 2021. Plants were grown in the research greenhouse with RH around 75%, temperature at 18–32C in soilless media with drip irrigation. All plants were fertigated daily with a solution containing macro‐ (N, 162; P, 30; K, 222; Ca, 136; Mg, 62; S, 100 ppm) and micronutrients (Fe, 1.0; Mn, 0.45; B, 0.1; Zn, 0.33; Cu, 0.035; Mo, 0.01; and NH_4_, 8.2 ppm), via an individual emitter in each pot. Flowering began in late June to early July 2021 and pollination was conducted by hand using a fine paintbrush early in the morning when flowers were open. Fruits were harvested in October and November (Figure 2).

### 2.3 Isolating endophytic fungi from the fresh tissues of monk fruit seedlings and mature plants

Samples of roots, stems, and leaves from five seedlings (9–10 weeks old) in the growth chamber were taken for endophyte isolation following the methods described by Musa et al. (2023). Small pieces (about 0.5 cm × 0.5 cm in size) of plant tissue were surface sterilized and rinsed with sterile reverse osmosis (RO) water using the method described above for isolation of seed endophytes. Fungal hyphae emerging from the tissue were selected and transferred repeatedly to PDA+ 50 ppm streptomycin to obtain a pure culture. Endophytes were isolated from leaves (young and old), stems (young and old), roots (from bulb and roots in soil), flowers (buds and fully‐open flowers), and fruit (pulp, seeds, and skin separated) at different maturity stages from 17 mature monk fruit plants grown in the greenhouse (Table 1). The isolation and purification procedures were the same as for seeds and seedlings described above.

**TABLE 1:** Number of samples collected from 17 fruiting monk fruit plants grown in the research greenhouse at the Institute for Sustainable Horticulture, KPU in 2021.

Leaves	Flowers	Fruit	Stems	Roots	Total samples
71	60	15	35	72	253

### 2.4 Identifying endophytic fungi

After pure cultures of endophytes were obtained, they were identified morphologically by microscopy and genetically by DNA sequencing. DNA was extracted using a protocol described by Cenis (1992) and subsequently amplified by polymerase chain reaction (PCR) using general internal transcribed spacer (ITS) primers, ITS1 and ITS 4 (White et al., 1990). The PCR products were sent for sequencing to Psomagen Inc., Rockville, MD, USA. The internal transcribed spacer (ITS) sequences of the endophytic fungi were compared to sequences deposited in GenBank using the National Centre for Biotechnology Information (NCBI) nucleotide basic local alignment search tool (BLASTn) (http://www.ncbi.nlm.nih.gov/BLAST). Isolates were identified to genus and species based on the highest % identity in BLASTn, and morphological characteristics obtained by microscopy. Where more than one identification was possible in GenBank, the genus or species was confirmed by microscopic comparison of fungal morphology to published descriptions. In a few cases where similar genera or species that could not be reliably resolved by BLAST analysis or microscopic examination, both names are shown. Subsequently, each fungal taxon was classified using the NCBI taxonomy browser database, US National Library of Medicine, Bethesda, MD (https://www.ncbi.nlm.nih.gov/taxonomy/browser/wwwtax.cgi). Fifty‐seven isolates from the mature plants that were less common, or had potential agronomic or other useful applications, have been stored in the Canadian Collection of Fungal Cultures (DAOMC) in Ottawa, ON, Canada, under specimen numbers 252740–252796.

### 2.5 Analysis of endophytic fungal communities

Non‐metric multidimensional scaling (NMDS) was carried out to explore the similarity of fungal communities among roots, stems, leaves, flowers, fruits, and seeds based on fungal orders (Peters et al., 2020). NMDS was performed using Python (3.9.16) (Van Rossum & Drake, 1995) with MDS implemented in the scikit‐learn (sklearn) library.

## 3 RESULTS

### 3.1 Overall fungal community composition

At least 150 species of fungal endophytes representing approximately 60 genera and 20 orders were recovered in culture from the monk fruit tissues. Twenty‐seven isolates of endophytic fungi were obtained from Chinese monk fruit seeds, either dry (purchased through Alibaba) or extracted from fresh fruit from China (Table 2). Another 22 isolates were obtained from seedlings grown from the fresh seeds (Table 3). The most common genus isolated from seeds and seedlings combined was *Trichoderma* (22 isolates: 7 or 8 species), followed by *Diaporthe* (4 isolates: 4 spp.) and *Aspergillus* (5 isolates: 3 spp.) from seeds, and *Penicillium* spp. (9 isolates: 4 spp. from seedlings; 2 from seeds). In contrast, only four isolates were obtained from seeds extracted from fresh fruit harvested in the greenhouse: one each of *Aspergillus fumigatus*, *Penicillium aethiopicum*, an unidentified *Penicillium* sp., and *Pseudogymnoascus pannorum* (Table 4).

**TABLE 2:** Identity of fungal endophytes recovered from dry monk fruit seeds and seeds from fresh fruit from China based on rDNA ITS sequence analyses and morphology.

Order	Name	# of isolates	GenBank accession #	% identity	Seed source
Eurotiales	*Aspergillus hiratsukae*	3	MK841469.1; MF773659.1	98.39; 99.82; 99.38	Alibaba^a^, Fruit^b^
	*Aspergillus pseudoglaucus*	1	KX258805.1	99.61	Alibaba
	*Aspergillus tennesseensis*	1	MT582757.1	100	Alibaba
Botryosphaeriales	*Botryosphaeria dothidea*	1	MN634011.1	100	Fruit
Glomerellales	*Colletotrichum brevisporum*	2	KY705054.1; LC379210.1	100; 100	Fruit
	*Colletotrichum qilinense*	1	MZ475126.1	98.81	Fruit
Diaporthales	*Diaporthe hongkongensis*	1	MW202983.1	99.25	Fruit
	*Diaporthe phaseolorum*	1	MN650843.1	100	Fruit
	*Diaporthe subclavata*	1	MT199841.1	100	Fruit
	*Diaporthe unshiuensis*	1	MW341297.1	100	Fruit
Pleosporales	*Exserohilum mcginnisii/E. rostratum*	1	MT337556.1/MK640580.1	98.8; 98.8	Fruit
Eurotiales	*Penicillium brevicompactum*	1	KX426968.1	99.81	Alibaba
	*Penicillium sumatraense*	2	OQ608602.1; MT529218.1	97.53; 98.51	Alibaba
Hypocreales	*Trichoderma atroviride*	7	MN634667.1 (4); MT341775.1 (2); MT023026.1	100	Alibaba
	*Trichoderma viride*	3	MN634490.1 (2); MN634664.1	100	Alibaba, Fruit

*Note*: The closest match in BLASTn to sequences deposited in GenBank and percent identity are shown.

^a^ Dry seeds purchased via Alibaba.

^b^ Fresh seeds extracted from fresh fruits from China.

**TABLE 3:** Identity of fungal endophytes recovered from leaves, stems, and roots of monk fruit seedlings grown from seed from China based on rDNA ITS sequence analyses and morphology.

Order	Name	# of isolates	GenBank accession #	% identity	Source
Hypocreales	*Beauveria bassiana*	1	MT441874.1	99.8	Stem
Eurotiales	*Chromocleista* sp.	1	MN644766.1	99.83	Stem
Mortierellales	*Mortierella* sp.	1	HE605241.1	100	Stem
Eurotiales	*Paecilomyces tabacinus*	1	LT548280.1	100	Root
Eurotiales	*Penicillium citrinum*	2	MN634531.1; MT597829.1	100; 100	Stem
	*Penicillium meleagrinum*	1	MF135516.1	99.82	Stem
	*Penicillium steckii*	1	OP615071.2	99.82	Stem
	*Talaromyces islandicus*	2	FR670311.1	89.96; 89.93	Root
Hypocreales	*Trichoderma afroharzianum*	4	MN644793.1	100; 99.83 (2); 99.66	Root; Stem
	*Trichoderma asperellum*	2	KY659051.1; LN846687.1	100; 99.82	Root
	*Trichoderma atroviridae*	2	MT604177.1; MT626716.1	100; 99.43	Stem
	*Trichoderma harzianum*	2	MT626717.1; MF078650.1	100; 99.65	Root; Leaf
	*Trichoderma harzianum/T. lixii*	1	MH339867.1/EF596951.1	100/100	Stem
	*Trichoderma* sp.	1	MK870660.1	100	Stem

*Note*: The closest match in BLASTn to sequences deposited in GenBank and percent identity are shown.

Three hundred and twenty‐five isolates of fungal endophyte were obtained in culture from the 17 mature plants grown in the greenhouse: 99 from reproductive tissues (flowers, fruit, and seeds) (Table 4) and 226 from vegetative tissues (leaves, stems, and roots) (Table 5). Not all of these isolates could be identified to species. Due to the large number of isolates of some genera, such as *Penicillium*, not all were submitted for ITS sequencing but were identified to genus by microscopic examination. The most common genera isolated from reproductive tissues were *Arthrinium/Apiospora* spp. (22 isolates; isolated equally from flowers and fruit), *Aspergillus* spp. (17 isolates), *Chaetomium* spp. (18 isolates), *Penicillium/Talaromyces* spp. (14 isolates), and *Coprinellus micaceus* (six isolates). *Coprinellus micaceus* was isolated frequently from leaf tissue also (six isolates), plus five isolates of *Coprinellus flocculosus* and two species of the closely related genus C*oprinopsis*: *Coprinopsis alnivora* (two isolates) and *Coprinopsis cinerea* (12 isolates). Other genera frequently isolated from leaves were *Alternaria* spp. (11 isolates, including three from roots), *Aspergillus* spp. (13, including one *Asp. ochraceus* from roots), *Botrytis cinerea* (six), *Chaetomium* spp. [eight, including one isolate from a stem and two *Ch. aureum* (teleomorph: *Arcopilus aureus*) from roots], *Cladosporium* spp. (12), *Epicoccum nigrum* (seven, including one from a root), and *Hypoxylon* (18: 8 *H. macrocarpum* and 10 *H. rubiginosum*). Twenty‐seven isolates of *Penicillium* spp. were obtained, 13 from leaves and 14 from roots. Of the 11 isolates of *Plectosphaerella* obtained, nine were *Pl. oligotrophica* and two *Pl. cucumerinum*; all were from roots except one from a stem. Genera isolated frequently only from roots included *Fusarium* (13 isolates, including 10 *F. oxysporum* and one *F. haematococcum/F. solani*), *Paraphaeosphaeria sporulosa* (five), *Sarocladium kiliense/S. strictum* (11), *Simplicillium* spp. (five), and *Trichoderma* spp. (five). Only three fungal endophytes were obtained in 35 samples from mature plant stems: one isolate each of *Chaetomium globosum*, *Plectosphaerella oligotrophica*, and *Phialemonium inflatum*. In addition to *Coprinellus micaceus*, species isolated from both reproductive and vegetative tissues were *Acremonium* spp., *Amorphotheca resinae, Arthrinium* spp. and *Apiospora kogelbergensis*, *Aspergillus fumigatus* and *Asp. ochraceus*, *Beauveria bassiana* (three from leaves and four from fruit skin), *Chaetomium globosum*, *Cladosporium* spp., *Epicoccum nigrum* (one from fruit skin), *Penicillium citrinum/P. steckii* and other *Penicillium* and *Talaromyces* spp. Many other endophytic fungi were isolated only once from mature monk fruit plant tissues. Four isolates produced no match to ITS sequences in GenBank at the genus or species level and could be identified only as members of the Lasiosphaeriaceae or Pleosporales.

**TABLE 4:** Identity of fungal endophytes recovered from flowers, flower buds, fruits, and seeds of mature monk fruit plants grown in the KPU research greenhouse based on rDNA ITS sequence analyses and morphology.

Order	Name	# of isolates	GenBank accession #	% identity	Source	DAOMC ID #
Hypocreales	*Acremonium sclerotigenum/Scopulariopsis gossypii*	1	OQ207544.1/KU523862.1	99.81/99.81	Fruit pulp	
Pleosporales	*Alternaria* sp.	1	Morphology only	—	Fruit skin	
	*Alternaria alternata*	1	MK518438.1	99.43	Flower	
Heliotiales	*Amorphotheca resinae*	1	MN242723.1	97.86	Flower bud	
Xylariales	*Apiospora kogelbergensis*	1	OW982982.1	99.25	Fruit skin	252751
Xylariales	*Arthrinium* spp.	20	KX378907.1; KX378907.1	99.63; 96.71	Flower (7); Fruit pulp (3); Fruit skin (10)	
	*Arthrinium phaeospermum/Apiospora rasikravandrae*	1	GU266274.1/OP237040.1	99.65/99.47	Fruit skin	252772
Eurotiales	*Aspergillus fumigatus*	6	Morphology to leaf isolates MT529448.1; MT529125.1; MH793851.1	—	Fruit pulp (4); Fruit skin (1); Seed (1)	
	*Aspergillus ochraceus*	8	MN533721.1; MT447480.1; MN533721.1	99.62; 99.81; 99.63	Flower (1); Flower bud (1) Fruit pulp (1); Fruit skin (5)	
	*Aspergillus septulus*	1	MH861876.1	99.82	Fruit skin	252758
	*Aspergillus tamarii*	2	MH345899.1	99.12	Fruit skin	
Hypocreales	*Beauveria bassiana*	4	MT111139.1	99.62	Fruit skin	
Helotiales	*Botrytis cinerea*	1	OP794013.1	99.8	Fruit skin	
Sordariales	*Chaetomium* spp.	9	Morphology only	—	Flower (1); Fruit skin (8)	
	*Chaetomium cochliodes*	1	MT520580.1	99.03	Fruit skin	252796
	*Chaetomium globosum*	5	KY132166.1; KP067224.1	98.43; 99.81	Flower (1); Fruit skin (4)	252743
	*Chaetomium novozelandicum*	3	MZ724883.1	96.7	Fruit skin	
Cladosporiales	*Cladosporium* spp.	4	Morphology only	—	Flower bud (3); Fruit pulp (1)	
Agaricales	*Coprinellus micaceus*	6	LR961895.1; MF156262.1; MH855975.1; LR961895.1	99.69; 99.08; 99.7; 98.06	Flower (3); Flower bud (3)	
Agaricales	*Crustomyces* sp.*/C. subabruptus*	1	MN905889.1/MK454922.1	99.67/99.50	Fruit skin	252789
Pleosporales	*Epicoccum nigrum*	1	FM200455.1	99.4	Fruit skin	
Hypocreales	*Fusarium graminearum*	1	KJ017740.1	99.59	Flower	252744
Saccharomycetales	*Hyphopichia burtonii*	1	MG554248.1	99.75	Fruit skin	
Eurotiales	*Paecilomyces variotii*	1	OW988300.1	99.47	Fruit skin	
Eurotiales	*Penicillium* spp.	5	Morphology only	—	Flower (1); Fruit pulp (1); Fruit skin (2); Seed (1)	
	*Penicillium aethiopicum*	1	ON428665.1	98.89	Seed	252742
	*Penicillium citrinum /P. steckii*	1	MG554368.1/KX610136.1	99.82/99.64	Fruit skin	252748
	*Penicillium glabrum /P. corylophilum*	1	MT797199.1/MT441635.1	99.44/99.44	Flower bud	252741
Polyporales	*Phlebia tremellosa*	1	OL436998.1	99.7	Flower bud	
*Incertae sedis*	*Pseudogymnoascus pannorum*	1	KF156305.1	99.61	Seed	252762
Chaetothyriales	*Rhinocladiella similis*	1	MH063252.1	100	Flower	
Eurotiales	*Talaromyces* sp.	5^a^	MK450749.1	99.29/97.7	Fruit skin	
	*Talaromyces pupureogenus*	1	MT635321.1	99.81	Fruit skin	252747
Polyporales	*Trametes hirsuta*	1	MF161297.1	99.66	Flower	252776

*Note*: The closest match in BLASTn to sequences deposited in GenBank and percent identity are shown, and the specimen ID # of isolates deposited in the Canadian Collection of Fungal Cultures (DAOMC).

^a^ All five isolates were the same *Talaromyces* species; no specific ID in GenBank.

**TABLE 5:** Identity of fungal endophytes recovered from leaves, stems, and roots of mature monk fruit plants grown in the KPU research greenhouse based on rDNA ITS sequence analyses and morphology.

Order	Name	# of isolates	GenBank accession #	% identity	Source	DAOMC ID #
Hypocreales	*Acremonium roseolum*	1	MH858153.1	98.66	Leaf	
	*Acremonium hyalinulum*	1	KP131521.1	98.68	Leaf	
Pleosporales	*Alternaria alternata*	2	OP696965.1; OL711657.1	99.61; 99.62	Leaf (1); Root bulb (1)	
	*Alternaria infectoria*	1	MK801346.1	95.15	Leaf	252756
	*Alternaria* spp.	8	OK274326.1; OK274326.1; KX139150.1; MK640587.1; MW534563.1; HQ649962.1	100; 99.46; 84.0; 99.5; 98.67; 99.38	Leaf (6); Root bulb (2)	
Heliotiales	*Amorphotheca resinae*	2	MN242723.1; KJ207403.1	98.34; 95.96	Leaf (2)	252753
Xylariales	*Apiospora kogelbergensis*	1	OW982982.1	99.25	Leaf	
Xylariales	*Arthrinium* spp.	4	KX378907.1; KX148691.1	100; 99.24	Leaf (4)	
Orbiliales	*Arthrobotrys amerospora*	1	KU702707.1	99.83	Root hair	
Eurotiales	*Aspergillus flavipes*	1	MN956655.1	99.62	Leaf	252764
	*Aspergillus fumigatus*	7	MT529448.1; MT529125.1; MH793851.1	98.92; 99.28 95.01	Leaf (6)	
	*Aspergillus ochraceus*	4	Morphology to flower/fruit isolates MN533721.1; MT447480.1; MN533721.1	—	Leaf (3); Root hair (1)	
	*Aspergillus tamarii*	1	MK332591.1	97.99	Leaf	252759
Dothidiales	*Aureobasidium pullulans*	1	MT645930.1	93.0	Leaf	252754
Hypocreales	*Beauveria bassiana*	3	OK331343.1	98.46	Leaf	
Hypocreales	*Bionectria* sp. (anamorph: *Clonostachys* sp.)	2	MH729023.1; KU951245.1	99.61; 99.67	Root bulb (1); Root hair (1)	
Saccharomycetales	*Blastobotrys* sp.	1	MK246187.1	99.81	Root bulb	
Helotiales	*Botrytis cinerea*	6	OM349592.1; MT150132.1; MH992148.1; AB693927.1; MK513827.1; MF661902.1	100; 100; 99.39; 100; 100; 88.25	Leaf	
Cephalothecales	*Cephalotheca sulfurea*	1	OM262341.1	99.61	Leaf	252787
Microascales	*Cephalotrichum purpureofuscum/Doratomyces* sp.		OP038661.1/KU954345.1	99.29/99.47	Leaf	252788
Sordariales	*Chaetomidium leptoderma*	4	NR_164219.1; JN573175.1	97.61; 97.68; 97.86	Root bulb (3); Root hair (1)	252763
Sordariales	*Chaetomium aureum* (teliomorph: *Arcopilus aureus*)	2	KP278194.1; MW533023.1	100; 100	Root bulb	252779
	*Chaetomium globosum*	3	KP067223.1; MF476072.1	100; 98.21	Leaf (2); Stem (1)	252766; 252775
	*Chaetomium novozelandicum*	2	MZ724883.1; MZ724884.1	98.82; 99.81	Leaf	252765
	*Chaetomium spinosum*	1	MH861746.1	99.05	Leaf	
Cladosporiales	*Cladosporium herbarum*	1	ON712476.1	99.4	Leaf	
	*Cladosporium ramotenellum*	1	OP006753.1	99.8	Leaf	
	*Cladosporium tenuissimum*	1	MK905459.1	99.39	Leaf	
	*Cladosporium* spp.	9	ON208763.1; KT826671.1; MH137774.1	93.16; 98.99; 98.39	Leaf	
Agaricales	*Coprinellus flocculosus*	5	MK656240.1	96.88; 96.88; 97.31	Leaf	
	*Coprinellus micaceus*	6	MF156262.1; MF156262.1; LR961895.1; LR961895.1; MF156262.1; LR961895.1	100; 99.84; 99.84; 99.23; 99.84; 98.06	Leaf	
Agaricales	*Coprinopsis alnivora*	2	MZ407758.1	98.02	Leaf	
	*Coprinopsis cinerea*	12	MN841919.1; MF351861.1; MN841919.1; MN841919.1	96.91; 99.68; 99.69; 99.85	Leaf	
Pleosporales	*Curvularia canadensis /C. inaequalis*	1	NR_170004.1; OK117928.1	99.62; 99.62	Leaf	
	*Curvularia coatesiae*	1	LC605635.1	96.53	Leaf	252755
Diaporthales	*Diaporthe eres*	1	MK335735.1	99.63	Root bulb	252770
Pleosporales	*Didymella anserina*	1	MN612779.1	99.14	Leaf	252740
Pleosporales	*Epicoccum nigrum*	7	OP315769.1; OP315769.1; OP315769.1; MH861752.1	100; 100; 99.59; 99.79	Leaf (6); Root bulb (1)	
Polyporales	*Fomitopsis mounceae*	1	MH086786.1	98.03	Leaf	
Hypocreales	*Fusarium haematococcum/F. solani*	1	MH729023.1/KU951245.1	99.61/99.67	Root hair	252778
	*Fusarium lichenicola*	1	KM921661.1	99.42	Root bulb	
	*Fusarium oxysporum*	10	KR906700.1; KC304797.1; FJ824032.1; MT529814.1	95.63; 99.8; 99.8; 98.29	Root bulb (6); Root hair (4)	
	*Fusarium tricinctum*	1	MN833356.1	99.81	Root bulb	252752
Xylariales	*Hypoxylon macrocarpum*	8	HM192912.1	96.38; 99.46; 98.6; 98.43; 99.3; 98.78; 97.69; 99.47	Leaf	
	*Hypoxylon rubiginosum*	10	AY787708.2; MT214998.1	99.80; 99.80; 99.80; 99.41; 99.80; 100; 91.2; 92.88	Leaf	252769; 252773
Sordariales	Lasiosphaeriaceae	3	KX343155.1; MN541090.1	99.8; 99.59	Root hair	252774
Mortierellales	*Linnemannia zychae*	1	MH857054.1	99.83	Root bulb	
Pleosporales	*Lophiostoma corticola /Angustimassarina coryli*	1	MK907710.1/MF167431.1	100/100	Leaf	252794
Mortierellales	*Mortierella hyalina*	1	MT003063.1	99.83	Root bulb	
Xylariales	*Nemania* sp.	1	MT153669.1	99.22	Leaf	252749
Xylariales	*Nigrospora oryzae*	1	KC131293.1	99.41	Leaf	252757
Agaricales	*Panaeolus subbalteatus*	1	MH855553.1	98.73	Leaf	252791
Pleosporales	*Paraconiothyrium fuckelii*	1	MK052700.1	99.29	Leaf	252792
Pleosporales	*Paraphaeosphaeria sporulosa*	5	KX302013.1; MH859903.1	99.82; 99.82	Root bulb (4); Root hair (1)	252761; 252771
Eurotiales	*Penicillium canescens*	1	MH865756.1	99.62	Leaf	
	*Penicillium cataractarum /P. simplicissimum*	2	MK534497.1/KM613146.1; MK534497.1/MT303132.1	99.44/100; 99.63/99.45	Root bulb (1); Root hair (1)	252767
	*Penicillium cinnamopurpureum*	1	MH655003.1	99.65	Leaf	252746
	*Penicillium citrinum/P. steckii*	1	KX610174.1/MT582790.1	95.16/94.72	Root bulb	
	*Penicillium* spp.	22	OP035353.1; OP647345.1; KY401082.1; MH512953.1; MH512953.1; MH512953.1; ON182131.1; ON182131.1; ON182131.1; ON182131.1	99.62; 99.06; 99.44; 99.26; 99.45; 99.26; 100; 100; 99.81; 99.81	Leaf (11); Root bulb (11)	
Pleosporales	*Periconia byssoides*	1	MK907734.1	99.63	Leaf	252795
Pleosporales	*Phaeosphaeria* sp.	1	ON520767.1	99.6	Leaf	252785
Cephalothecales	*Phialemonium inflatum*	1	NR_165996.1; MH857776.1	99.61; 96.58	Stem	252783
Glomerellales	*Plectosphaerella cucumerinum*	2	ON927102.1; MW850542.1	99.4; 99.8	Root bulb	252777; 252790
	*Plectosphaerella oligotrophica*	9	MT447499.1	99.6; 99.8	Root bulb (3); Root hair (5); Stem (1)	252782; 252784
Pleosporales	Unidentified	1	MG916998.1	99.22	Leaf	252768
Hypocreales	*Purpureocillium lilacinum*	1	KJ862077.1	99.64	Leaf	
Hypocreales	*Sarocladium kiliense/S. strictum*	11	KX384658.1/MF077236.1; KX384658.1/MF077236.1; KF293986.1/MF077237.1; KF293986.1/ON500613.1	99.44/99.25; 99.81/99.62; 99.62/99.43; 100/99.81	Root bulb (9); Root hair (2)	
Agaricales	*Schizophyllum commune*	1	ON500589.1	99.83	Leaf	
Microascales	*Scopulariopsis brevicaulis*	1	OW987158.1	99.83	Leaf	
Hypocreales	*Simplicillium aogashimaense*	3	AB604004.1; MK579181.1; MK579181.1	99.63; 99.28; 99.27	Root bulb	252780; 252781
	*Simplicillium obclavatum*	1	KC403970.1	91.9	Root bulb	252786
	*Simplicillium subtropicum*	1	MW260103.1	99.47	Root bulb	252750
Sordariales	*Sordaria fimicola*	1	JX273473.1	99.26	Leaf	
Hypocreales	*Trichoderma ghanense*	1	MT520628.1	96.0	Root bulb	252745
	*Trichoderma* spp.	4	Morphology only	—	Root bulb (1); Root hair (3)	
Ustilaginales	*Ustanciosporium appendiculatum*	1	GQ888733.1	91.12	Leaf	252760
Helotiales	*Varicosporium delicatum*	1	JQ412864.1	93.75	Leaf	252793

*Note*: The closest match in BLASTn to sequences deposited in GenBank and percent identity are shown, and the specimen ID # of isolates deposited in the Canadian Collection of Fungal Cultures (DAOMC).

### 3.2 Fungal community by plant part

**FIGURE 3:**
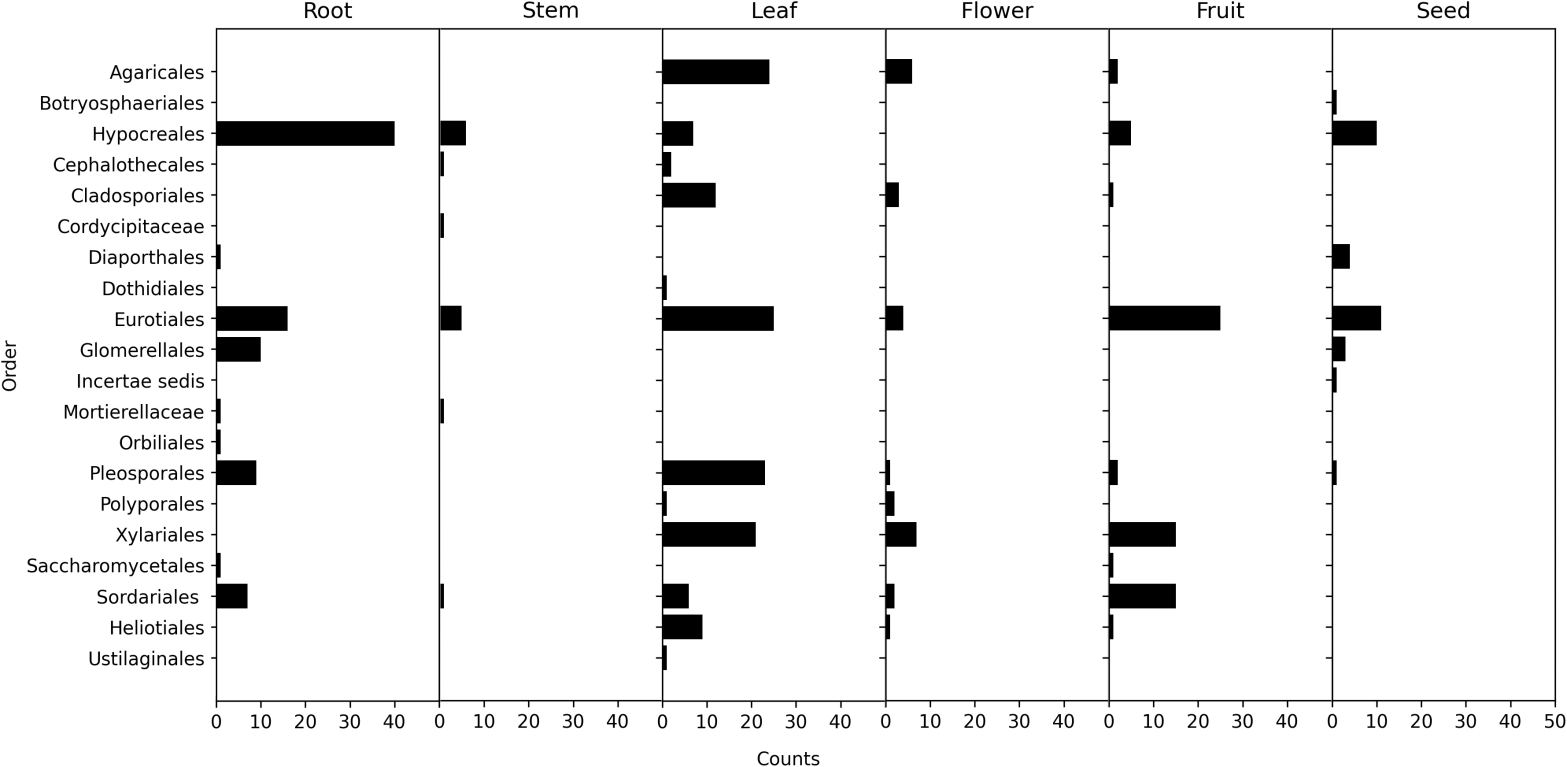
Number of fungal isolates in different taxonomic orders isolated from roots, stems, leaves, flowers, fruits, and seeds of monk fruit.

**FIGURE 4:**
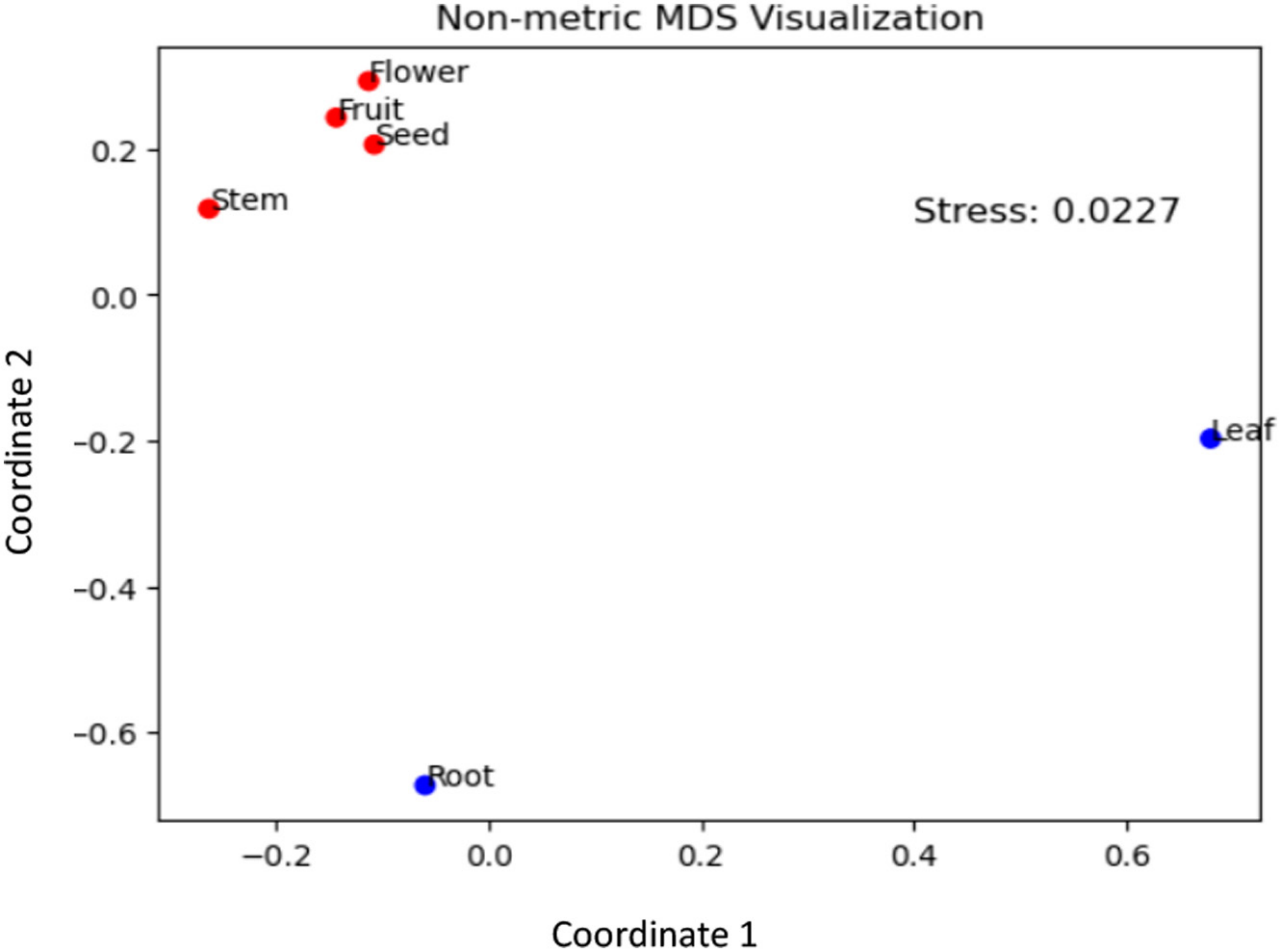
Measure of dissimilarity in the endophytic fungi composition among the root, stem, leaf, flower, fruit, and seed of monk fruit using non‐metric multidimensional scaling.

Fungal community composition differed among roots, stems, leaves, flowers, fruits, and seeds (Figures 3 and 4). The combined isolates represented 20 taxonomic orders. The dominant orders across all plant parts were Eurotiales (24%), Hypocreales (19%), and Pleosporales (10%) (Figure 3). Leaves (12 orders) had the greatest diversity and abundance of fungal endophytes, followed by roots (nine orders), fruits (nine orders), flowers (eight orders), seeds (seven orders), and stems (six orders) (Figure 3). The dominant orders were Eurotiales (25 isolates), Agaricales (24 isolates), Pleosporales (23 isolates), and Xylariales (21 isolates) in leaves and Hypocreales (40 isolates), Eurotiales (16 isolates), and Glomerellales (10 isolates) in roots. The dominant orders in flowers, fruits, and seeds were Eurotiales (40 isolates), Xylariales (22 isolates), Sordariales (17 isolates), and Hypocreales (15 isolates), followed by Agaricales (eight isolates). The NMDS (stress = 0.0227) analysis showed the similarity/dissimilarity in fungal community composition among different plant parts (Figure 4). The root and leaf fungal communities showed a strong distinction from each other and those of the reproductive plant parts (flowers, fruits, and seeds), which were more similar in their endophyte composition. The six orders of fungal endophytes isolated from stems were more similar to the communities found in the reproductive tissues (flowers, fruits, and seeds) than to those in the leaves or roots. Some of the endophytic isolates could have originated horizontally, that is, from the greenhouse environment, rather than vertically from within the monk fruit plants themselves since the greenhouse was not completely isolated from the outdoor environment and the soilless media was not sterile.

## 4 DISCUSSION

Monk fruit plants proved to be a rich source of fungal endophytes with a great diversity and abundance, especially in leaves. The role of these fungi in the monk fruit plants is likely to be as complex as their diversity. Some may be neutral commensalists, while others, such as the wood‐decaying Xylariaeceae (*Hypoxylon*, *Nemania*), Meruliaceae (*Phlebia tremellosa*), Psathyrellaceae (*Coprinellus* and *Coprinopsis* spp.), and Polyporaceae (*Trametes hirsuta*), may play a beneficial role in vegetative decay and nutrient cycling in the natural environment, or protection against pathogens or herbivores. Members of the Xylariales, in particular, produce a wide array of secondary metabolites many of which are antagonists of other fungi and bacteria (Becker & Stadler, 2021). A few of the species isolated may be hyperparasites of other fungal endophytes found in the monk fruit tissues, for example, *Penicillium [Eupenicillium] cinnamopurpureum* which grows on the heads of *Aspergillus* spp. (Horn & Peterson, 2008).

In addition to the Xylariales, many of the other fungal species obtained from the monk fruit plants are known to produce bioactive compounds with medical or industrial applications. For example, *Talaromyces purpureogenus* (Keekan et al., 2020) and *Penicillium brevicompactum* (Fonseca et al., 2022) produce pigments with commercial applications in the food processing industry. Several species are known to produce antibiotics, such as diketopiperazine, produced by *Paraphaeosphaeria sporulosa*, which is effective against salmonella bacteria (Carrieri et al., 2020). *Panaeolus subbalteatus* is one of the most common sources of psilocybin, used in medical treatment. The kerosene fungus, *Amorphotheca resinae* (anamorph: *Hormoconis resinae*), which was isolated from both leaves and flower buds, damages jet fuel, diesel, petroleum and creosote‐treated wood, but may have useful environmental applications in remediation of hydrocarbon contaminated sites (Rafin & Veignie, 2018). *Chaetomium* spp. are the source of more than 100 useful secondary metabolites (Dwibedi et al., 2023). For example, *Arcopilus aureus* (anamorph: *Chaetomium aureum*) produces high levels of resveratrol, a potent antioxidant, and sclerotiorin, which has anti‐cancer properties (Dwibedi & Saxena, 2018). *A. aureus* has high lead tolerance and clearance, suggesting a potential role in bioremediation of contaminated soils (Da Sila et al., 2018).

Several of the endophytic species obtained in this study have potential agricultural applications in enhancing plant growth and tolerance to drought and other environmental stresses, or as biological control agents of disease and insect pests. The abundance and diversity of the fungal endophytes recovered from the monk fruit plants suggest multiple, layered means of protection against potential pests and adaptation to environmental stresses. Many endophytic species with anti‐fungal or plant growth‐promoting activity recovered in this study have also been isolated from grapevines (*Vitis vinifera* L.) (Kulišová et al., 2021), including species of *Aspergillus, Alternaria, Chaetomium, Epicoccum*, and *Penicillium*. These and several other species isolated from leaves and fruit skin, are also common epiphytes that play a role in crop protection both on and below the leaf surface, and are often transmitted horizontally. In grape, the most effective antifungal endophytes against *Botrytis cinerea*, the cause of bunch rot, were *Alternaria* and *Epicoccum* species which, along with *Aspergillus fumigatus*, produce high levels of siderophores and antioxidants (Kulišová et al., 2021). Endophytic strains of *E. nigrum* have been shown to reduce the incidence and severity of a range of plant diseases (Taguiam et al., 2021). In British Columbia, an isolate of *E. nigrum* from mummy berry‐infected blueberries suppressed spring apothecia production of *Monilinia vaccinii‐corymbosi* when applied to soil after infected berries dropped (Kitura et al., 2023). *Hypoxylon rubiginosum* has shown promise as a biocontrol for dieback of European ash (*Fraxinus excelsior* L.), associated with its production of the anti‐fungal metabolite, phomopsidin (Halecker et al., 2020). *Simplicillium aogashimaense* and *S. obclavatum*, isolated here from monk fruit root bulbs, are mycoparasites that have shown efficacy against, respectively, powdery mildew and stripe rust of wheat (Wang et al., 2020; Zhu et al., 2022). *Paecilomyces variotii* is an effective biocontrol agent of gummy stem blight and powdery mildew of cucumber, and has been shown to inhibit other plant pathogens including nematodes (Moreno‐Gavíra et al., 2021). *Purpureocillium lilacinum* [syn. *Paecilomyces lilacinus* (Thom) Samson] is a parasite of nematode eggs (Kiewnick & Sikora, 2004), an entomopathogen, and has been shown to promote the growth of tomato under heavy metal stress (Musa et al., 2023). Strains of *P. lilacinum* have been registered in the USA and Europe for control of parasitic nematodes in crops. *Arthrobotrys amerispora*, isolated from a root hair of the monk fruit, may be playing a role in root protection; *Arthrobotrys* spp. are well‐known nematode‐trapping fungi as well as mycoparasites (Gams et al., 2004). Eight endophytic strains of the entomopathogen *Beauveria bassiana* were recovered from the monk fruit tissues, in addition to a *Bionectria* sp. (anamorph: *Clonostachys*; syn. *Gliocladium*) and several *Trichoderma* spp., which are well‐known protectors of plants from pathogen and insect attack, as well as plant growth promoters (Sharma & Gothalwal, 2017).

For some plant pathogenic fungi, existence as an endophyte may be a latent stage in pathogenesis. Disease develops as the host plant reaches a certain life stage or begins to senesce, or as the plant experiences environmental stress or other damage. *Botrytis cinerea*, for example, is a common pathogen causing gray mold disease of many crops but is often found as an endophyte in healthy plant tissues. The two *Colletotrichum* spp. isolated from the internal tissues of monk fruit seeds in this study are known plant pathogens and may be a quiescent stage in the development of anthracnose disease. *Plectosphaerella cucumerinum* (syn. *Plectosporium tabacinum*) causes wilt and root rot of several crops including cucurbits, tomato, potato, and basil (Raimondo & Carlucci, 2018) and may be a quiescent pathogen in the monk fruit plants, while *Pl. oligotrophica* is a low‐carbon feeding, soil saprophyte (Liu et al., 2013) that may be neutral, or play a beneficial role in the presence of biotic or abiotic stresses. As an example of the multiple potential roles of a single endophytic species, *Pl. cucumerinum* is also nematophagous and has been tested for biocontrol of potato cyst nematode (Atkins et al., 2003), although, more recently, it has also been shown to cause potato wilt disease in China (Gao et al., 2016) and Pakistan (Alam et al., 2021). *Paraconiothyrium fuckelii* (syn. *Leptosphaeria coniothyrium*, basionym: *Coniothyrium fuckelii*) is a wound pathogen causing cane blight of raspberry, rose, and other woody hosts worldwide (Guarnaccia et al., 2022). It is also known as a saprobe, but its potential role as an endophyte in these hosts has not been explored.

Among some species of plant pathogens, endophytic and pathogenic strains have quite different relationships and effects on their hosts. Endophytic strains of *Fusarium oxysporum* have been shown to reduce root rot and wilt diseases caused by pathogenic strains in tomato and other crops (de Lamo & Takken, 2020). The endophytic strains of *F. oxysporum* have fewer effectors and exhibit different patterns of tissue colonization and triggering of host defenses than pathogenic strains. Further understanding of the role of endophytes in plant protection and pathogenesis may reveal additional new, sustainable methods of plant disease control.

In summary, monk fruit plants can be easily grown in the greenhouse and are a prolific source of endophytic fungi and secondary metabolites for potential research and development. This work has deepened our understanding of the intricate interactions between plants and fungi that sustain ecosystems and underpin plant health and resilience. These findings can inform strategies for developing climate‐resilient crops and restoring ecosystems in the face of climate challenges and developing more sustainable and eco‐friendly strategies for plant health management. Our analysis did not include bacterial or viral endophytes, or fungi that did not grow on PDA. Further investigation of monk fruit as a potential source of these endophytes may reveal even more useful strains and advance our understanding of how endophytes interact with their hosts.

## CONFLICT OF INTEREST STATEMENT

The authors declare no conflict of interest.

## Data Availability

The data that support the findings of this study are openly available in figshare: 10.6084/m9.figshare.24530542.
